# Endothelial Progenitor Cell Migration-Enhancing Factors in the Secretome of Placental-Derived Mesenchymal Stem Cells

**DOI:** 10.1155/2016/2514326

**Published:** 2016-01-06

**Authors:** Witchayaporn Kamprom, Pakpoom Kheolamai, Yaowalak U-Pratya, Aungkura Supokawej, Methichit Wattanapanitch, Chuti Laowtammathron, Sittiruk Roytrakul, Surapol Issaragrisil

**Affiliations:** ^1^Department of Immunology, Faculty of Medicine Siriraj Hospital, Mahidol University, Bangkok 10700, Thailand; ^2^Siriraj Center of Excellence for Stem Cell Research, Faculty of Medicine Siriraj Hospital, Mahidol University, Bangkok 10700, Thailand; ^3^Division of Cell Biology, Faculty of Medicine, Thammasat University, Pathumthani 12120, Thailand; ^4^Center of Excellence in Stem Cell Research, Faculty of Medicine, Thammasat University, Pathumthani 12120, Thailand; ^5^Division of Hematology, Department of Medicine, Faculty of Medicine Siriraj Hospital, Mahidol University, Bangkok 10700, Thailand; ^6^Department of Clinical Microscopy, Faculty of Medical Technology, Mahidol University, Bangkok 73170, Thailand; ^7^Proteomics Research Laboratory, Genome Institute, National Science and Technology Development Agency, Pathumthani 12120, Thailand

## Abstract

Therapeutic potentials of mesenchymal stem cells (MSCs) depend largely on their ability to secrete cytokines or factors that modulate immune response, enhance cell survival, and induce neovascularization in the target tissues. We studied the secretome profile of gestational tissue-derived MSCs and their effects on functions of endothelial progenitor cells (EPCs), another angiogenic cell type that plays an important role during the neovascularization. MSCs derived from placental tissues (PL-MSCs) significantly enhanced EPC migration while BM-MSCs, which are the standard source of MSCs for various clinical applications, did not. By using protein fractionation and mass spectrometry analysis, we identified several novel candidates for EPC migration enhancing factor in PL-MSCs secretome that could be used to enhance neovascularization in the injured/ischemic tissues. We recommend that the strategy developed in our study could be used to systematically identify therapeutically useful molecules in the secretomes of other MSC sources for the clinical applications.

## 1. Introduction

Mesenchymal stem cells (MSCs) are multipotent stem/progenitor cells which can differentiate to several mesodermal derivatives and possess an ability to secrete factors involved in neovascularization and immunomodulation [1, 2]. Previous studies demonstrated that MSCs could ameliorate the pathology associated with ischemic heart disease, ischemic stroke, and peripheral vascular disease by releasing several critical factors that increase cell survival, provide an appropriate microenvironment for repairing damaged tissue, and induce neovascularization [3–5].

For several decades, neovascularization is believed to be accomplished by proliferation of mature endothelial cells residing in the local vessels through the process of angiogenesis [6]. However, recent studies have demonstrated that other angiogenic cells called endothelial progenitor cells (EPCs) also play an important role during the formation of new vessels through the process of vasculogenesis [7–9]. Vasculogenesis involves migration of EPCs from blood circulation into injured/ischemic tissues where they proliferate and generate new vessels* de novo*. The effects of factors released from bone-marrow-derived MSCs (BM-MSCs), which are the standard source of MSCs for clinical applications, on the functional properties of mature endothelial cells have been previously reported [10–12]; however the effects of MSC-derived factors on EPC functions have yet to be characterized.

Apart from BM-MSCs which require highly invasive procedure for their isolation, MSCs can be easily obtained in large quantity from several gestational tissues using noninvasive procedure [13, 14]. Gestational tissues have currently been considered more suitable sources of MSCs for clinical applications. However, recent studies showed that MSCs derived from distinct sources might produce distinct combination of factors that exert different effects on target cells [15, 16]. Therefore, characterization of the secretome of distinct MSC sources is critical for selecting suitable MSCs for specific applications. The secretomes of BM-MSCs and adipose-derived MSCs were recently studied [17–19]; however the secretomes of gestational tissue-derived MSCs are still poorly characterized.

The present study aimed to investigate the effect of factors released from placental-derived MSCs (PL-MSCs) on EPC migration, which is a critical step of vasculogenesis, using an* in vitro* migration assay. Moreover, the candidate EPC migration inducing factors presented in PL-MSCs secretome were identified using protein fractionation and mass spectrometry analysis.

## 2. Materials and Methods

### 2.1. Isolation and Culture of PL-MSCs

This study was approved by the Siriraj Institutional Review Board, Faculty of Medicine Siriraj Hospital, Mahidol University, which was in accordance with the Declaration of Helsinki, the Belmont Report, CIOMS Guidelines, and ICH-GCP. Placental tissues and umbilical cord blood were obtained from healthy newborns after receiving signed informed consents from their mothers. Placental tissues were minced into small pieces and incubated with 0.25% trypsin-EDTA at 37°C for 30 minutes in shaking water bath. After incubation, the digested tissues were plated into 25 cm^2^ tissue culture flask containing complete medium (Dulbecco's Modified Eagle's Medium (DMEM; GIBCO, Invitrogen Corporation, USA) supplemented with 10% (v/v) Fetal Bovine Serum (FBS; Lonza, USA), 100 U/mL penicillin (General Drug House Co., Ltd., Thailand), and 100 *μ*g/mL streptomycin (General Drug House Co., Ltd., Thailand)). The cultures were maintained at 37°C in a humidified atmosphere containing 5% CO_2_ and the medium was replaced every 3 days throughout the entire culture period.

### 2.2. Isolation and Culture of BM-MSCs

Bone marrow samples (*n* = 5) were obtained from healthy donors by bone marrow aspiration after giving a written informed consent. Bone marrow mononuclear cells (BM-MNCs) were isolated by density gradient centrifugation (400 g for 20 minutes at 20°C). The BM-MNCs were then resuspended in complete DMEM medium, plated into 25 cm^2^ flask at a density of 2 × 10^5^ cells/cm^2^, and cultured at 37°C in a humidified atmosphere containing 5% CO_2_. After 48-hour culture, the nonadherent cells were removed and fresh medium was added. Cultures were maintained at 37°C in a humidified atmosphere containing 5% CO_2_ and medium was replaced every 3 days throughout the entire culture period.

### 2.3. Immunophenotyping of PL-MSCs and BM-MSCs

The 3rd–5th passages of MSCs were harvested by trypsinization and incubated with 10 *μ*L of the following mouse anti-human antibodies: anti-CD34-FITC (BD Pharmingen, USA), anti-CD45-PE (BD Pharmingen, USA), anti-CD90-FITC (AbD Serotec, USA), anti-CD73-PE (BD Pharmingen, USA), and anti-CD105-PE (Miltenyi Biotec, Germany) for 30 minutes at 4°C in the dark. After incubation, cells were washed twice with PBS and fixed with 300 *μ*L 1% (v/v) paraformaldehyde. The expression profiles of cell surface markers were then determined by FACSCalibur flow cytometry (Becton Dickinson, USA) using CellQuest software. Cells labeled with FITC-conjugated mouse IgG1 (eBioscience, USA) and PE-conjugated mouse IgG1 (eBioscience, USA) served as negative controls.

### 2.4. Osteogenic and Adipogenic Differentiation of PL-MSCs and BM-MSCs

For adipogenic differentiation, 5 × 10^4^ MSCs (3rd–5th passages) were cultured in NH AdipoDiff Medium (Miltenyi Biotec, Germany). The medium was replaced every 3 days according to the manufacturer's instructions. After culture for 4 weeks, cells were stained with 0.5% (w/v) Oil Red O (Sigma Aldrich, USA) in isopropanol for 20 minutes at room temperature and were observed under phase-contrast microscope (Olympus, Japan). For osteogenic differentiation, 5 × 10^4^ MSCs (3rd–5th passages) were cultured in NH OsteoDiff Medium (Miltenyi Biotec, Germany). The medium was replaced every 3 days according to the manufacturer's instructions. After culture for 3 weeks, cells were stained with 40 mM Alizarin Red S (Sigma Aldrich, USA) for 20 minutes at room temperature and were observed under phase-contrast microscope (Olympus, Japan).

### 2.5. Culture and Characterization of EPCs from Umbilical Cord Blood

Twenty milliliters of heparinized umbilical cord blood was collected for EPC isolation. Mononuclear cell populations from umbilical cord blood were isolated using IsoPrep (Robbins Scientific Corporation, USA) density gradient centrifugation, washed twice with Phosphate Buffer Saline (PBS; GIBCO, Invitrogen Corporation, USA), resuspended in endothelial cell growth medium (endothelial basal medium-2 (LONZA, Germany), supplemented with EGM-2 single aliquots (LONZA, Germany) containing 2% (v/v) FBS, 5 *μ*g/mL epidermal growth factor, 200 *μ*g/mL hydrocortisone, 0.5 *μ*g/mL vascular endothelial growth factor, 10 *μ*g/mL basic fibroblast growth factor, 20 *μ*g/mL long R3 insulin-like growth factor 1, and 1 mg/mL ascorbic acid), and plated into an individual well of 6-well plate coated with 10 *μ*g/mL human fibronectin (Amersham Biosciences, USA) at a density of 1 × 10^6^ cells/well. After culture for 3 days, the nonadherent cells were removed and fresh medium was added. The cultures were maintained at 37°C in a humidified atmosphere containing 5% CO_2_ and the medium was replaced every 3 days throughout the entire culture period.

Cells were characterized for EPC surface markers by incubating with the following mouse anti-human antibodies: anti-CD34-FITC (R&D Systems, USA), anti-VEGFR2-PE (R&D Systems, USA), anti-CD31-PE (BioLegend, USA), and anti-vWF-FITC (R&D Systems, USA) for 15 minutes at 4°C in the dark. After incubation, cells were washed twice with PBS and fixed with 2% (v/v) paraformaldehyde in PBS. Flow cytometry was performed using FACSCalibur flow cytometer (Becton Dickinson, USA) and CellQuest software. Cells labeled with FITC-conjugated mouse IgG1 (eBioscience, USA) and PE-conjugated mouse IgG1 (eBioscience, USA) served as negative controls.

To further examine the characteristics of EPCs, an* in vitro* vessel formation assay was performed. Briefly, 100 *μ*L Matrigel (BD Bioscience, USA) was added to an individual well of 4-well plate (Corning, USA) and allowed to polymerization at 37°C for 1 hour. At this stage, 1 × 10^5^ EPCs were resuspended in endothelial cell growth medium, plated into an individual well of Matrigel-coated 4-well plate, and cultured at 37°C in a humidified atmosphere containing 5% CO_2_ for 24 hours. At the end of culture, the extent of capillary-like structure was observed by phase-contrast microscopy (Olympus, Japan).

### 2.6. Preparation and Fractionation of PL-MSCs Conditioned Medium

7 × 10^5^ PL-MSCs (3rd–5th passages) were plated into 75 cm^2^ flask containing complete medium and incubated in a humidified atmosphere containing 5% CO_2_ for 24 hours. After incubation, cells were washed twice with 5 mL sterile PBS and incubated with 15 mL serum-free medium (SFM) (DMEM supplemented with 100 U/mL penicillin and 100 *μ*g/mL streptomycin) for further 24 hours. After incubation, the PL-MSCs conditioned medium was collected, 15 mL fresh SFM was added, and cells were incubated for further 24 hours. After incubation, the conditioned medium was collected and pooled with the earlier collected PL-MSCs conditioned medium. The pooled PL-MSCs conditioned medium was then centrifuged at 400 g for 10 minutes at 4°C and filtered through 0.45 *μ*m syringe filter (Corning, USA). The filtered conditioned medium was fractionated into 5 distinct fractions according to the molecular weight of their protein composition using various ultraspin columns with molecular weight cut-off (MWCO) at 100 kDa (Pall Corporation, USA), 50 kDa (Merck, Germany), 30 kDa (Pall Corporation, USA), and 10 kDa (Pall Corporation, USA) according to the manufacturer's instructions. To fractionate PL-MSCs conditioned medium, the medium was transferred to 100 kDa ultraspin columns and centrifuged at 4700 g for 15 minutes at 4°C. After centrifugation, the fraction of PL-MSCs conditioned medium retained in the column was collected while the flow-through was transferred to the 50 kDa ultraspin columns for further centrifugation. By repeating this procedure with the 30 kDa and 10 kDa columns, the PL-MSCs conditioned medium was successfully fractionated into 100 kDa, 50 kDa, 30 kDa, 10 kDa, and less than 10 kDa fractions.

### 2.7. Preparation of the 100 kDa Subfraction of PL-MSCs Secretome by Reverse-Phase Chromatography

The 100 kDa fraction of PL-MSCs conditioned medium was further fractionated into several subfractions according to the hydrophobicity of their protein composition by reverse-phase chromatography. Firstly, Sep-Pak C18 Vac cartridge (Waters Associates, USA) was washed twice with 35 mL 100% acetonitrile (ACN) followed by equilibration with 35 mL 0.1% trifluoroacetic acid (TFA). The solutions were allowed to drain at the rate of 1 mL/min by connecting to Perista pump AC2110 (ATTO, Japan). After equilibration, the 100 kDa fraction was transferred into the preequilibrated Sep-Pak C18 cartridge followed by the addition of 35 mL 0.1% TFA. The fluid was allowed to drain and the hydrophobic proteins presented in the 100 kDa fraction were sequentially eluted from the column by increasing ACN concentration from 10% to 100%. The eluted solutions were collected and separated into distinct subfractions and the amount of proteins presented in each subfraction were determined by measuring an absorbance at 280 nm and 220 nm by nanodrop 2000 spectrophotometer (Thermo Scientific, USA). Finally, acetonitrile and TFA remaining in each subfraction were eliminated by vacuum centrifugation.

### 2.8. *In Vitro* Migration Assay

To investigate the paracrine effect of BM-MSCs and PL-MSCs on EPC migration, EPCs were cocultured with MSCs through 8 *μ*m pore size transwell (Corning, USA) as shown in Figure 3(a). Briefly, 5 × 10^4^ MSCs were plated into the lower chamber of transwell containing 600 *μ*L complete DMEM medium. The culture was then maintained at 37°C in a humidified atmosphere containing 5% CO_2_ for 24 hours to allow cell attachment. After 24-hour incubation, the medium was replaced with 600 *μ*L DMEM supplemented with 2% (v/v) FBS, 100 U/mL penicillin, and 100 *μ*g/mL streptomycin and cells were then cultured for further 24 hours. On the following day, 4 × 10^4^ EPCs were seeded into the upper chamber of transwell inserts which already contained MSCs in the lower chamber. The coculture was maintained at 37°C in a humidified atmosphere containing 5% CO_2_ for further 6 hours. At the end of culture, the number of EPCs which migrate to the other side of transwell inserts were determined by hematoxylin staining. The EPCs cultured in transwells whose lower chamber contained cell-free DMEM medium served as controls. Data were presented as mean ± SEM of three independent experiments.

The effect of fractionated PL-MSCs conditioned medium on EPC migration was studied using an* in vitro* migration assay. 4 × 10^4^ cells EPCs were seeded into the upper chamber of transwell inserts (Corning, USA) while the lower chamber was added with either 100 kDa, 50 kDa, 30 kDa, 10 kDa, or less than 10 kDa fraction of the conditioned medium. Cells were then incubated at 37°C in a humidified atmosphere containing 5% CO_2_ for further 6 hours. The migratory capacity of EPCs toward each fractionated PL-MSCs conditioned medium was determined by hematoxylin staining. EPCs cultured in transwells whose lower chamber contained serum-free medium (SFM) served as negative controls while EPCs cultured in transwells whose lower chamber contained unfractionated PL-MSCs conditioned medium served as positive controls. The effect of each 100 kDa subfraction on EPC migration was also determined by the same procedure as described above. In this case, EPCs cultured in transwells whose lower chamber contained unfractionated 100 kDa medium served as controls.

### 2.9. Mass Spectrometry Analysis and Protein Identification

Mass spectrometry analysis was performed at the National Center for Genetic Engineering and Biotechnology (BIOTEC), Thailand. The proteins presented in each PL-MSCs conditioned medium fraction were digested by incubation with trypsin and analyzed by ESI ion trap mass spectrometry. Identification and quantification of each protein was determined by DeCyder MS differential analysis software 2.0 (GE Healthcare, USA) and MASCOT search engine software (Matrix Science, UK) based on NCBInr human protein databases. The identified proteins were then categorized into nonsecretory, classical secretory, and nonclassical secretory proteins by SignalP and SecretomeP software (Center for Biological Sequence Analysis (CBS), Denmark). Finally, the secretory proteins were further categorized by PANTHER and UniProt software into separated groups according to their functions.

### 2.10. Statistical Analysis

Data were presented as mean ± standard error of the mean (SEM). The Mann-Whitney test and nonparametric Kruskal-Wallis test were used to assess the significance of differences between observed data. *P* < 0.05 was considered to be statistically significant.

## 3. Results

### 3.1. Characteristics of PL-MSCs, BM-MSCs, and CB-EPCs

**Figure 1:**
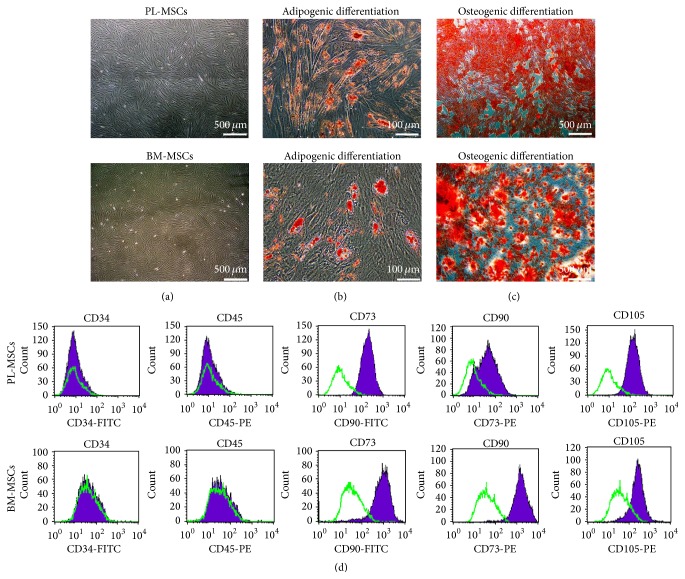
Characteristics of PL-MSCs and BM-MSCs. (a) Fibroblast-like morphology of PL-MSCs and BM-MSCs (scale bar = 500 *μ*m). (b) Adipogenic differentiation of PL-MSCs and BM-MSCs as demonstrated by Oil Red O staining (scale bar = 100 *μ*m). (c) Osteogenic differentiation of PL-MSCs and BM-MSCs as demonstrated by Alizarin Red S staining (scale bar = 500 *μ*m). (d) Immunophenotype of PL-MSCs and BM-MSCs as determined by flow cytometry.

**Figure 2:**
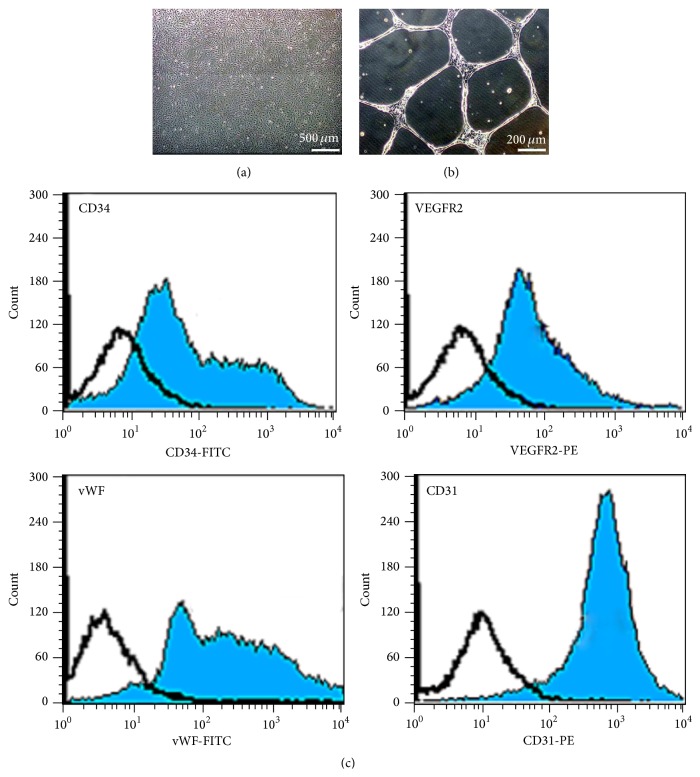
Characteristic of endothelial progenitor cells (EPCs). (a) Cobblestone-like morphology of EPC colony appeared after 7 days of culture (scale bar = 500 *μ*m). (b) Capillary-like structures derived from EPCs after culture on Matrigel for 24 hours (scale bar = 200 *μ*m). (c) Immunophenotype of EPCs as determined by flow cytometry.

Placenta-derived mesenchymal stem cells (PL-MSCs) and bone-marrow-derived mesenchymal stem cells (BM-MSCs) established in this study displayed fibroblast-like morphology (Figure 1(a)), could differentiate to adipocytes and osteocytes as demonstrated by Oil Red O (Figure 1(b)) and Alizarin Red S staining (Figure 1(c)), and exhibited typical MSC surface markers (positive for CD73, CD90, and CD105 and negative for hematopoietic markers CD34 and CD45) (Figure 1(d)). Umbilical cord blood-derived endothelial progenitor cells (CB-EPCs) established in this study displayed cobblestone-like morphology, which is typical for EPCs (Figure 2(a)), formed capillary-like structures on Matrigel (Figure 2(b)), and expressed typical EPC surface markers CD34, VEGFR2, vWF, and CD31 (Figure 2(c)).

### 3.2. Effect of MSC-Derived Secretory Factors on EPC Migration

**Figure 3:**
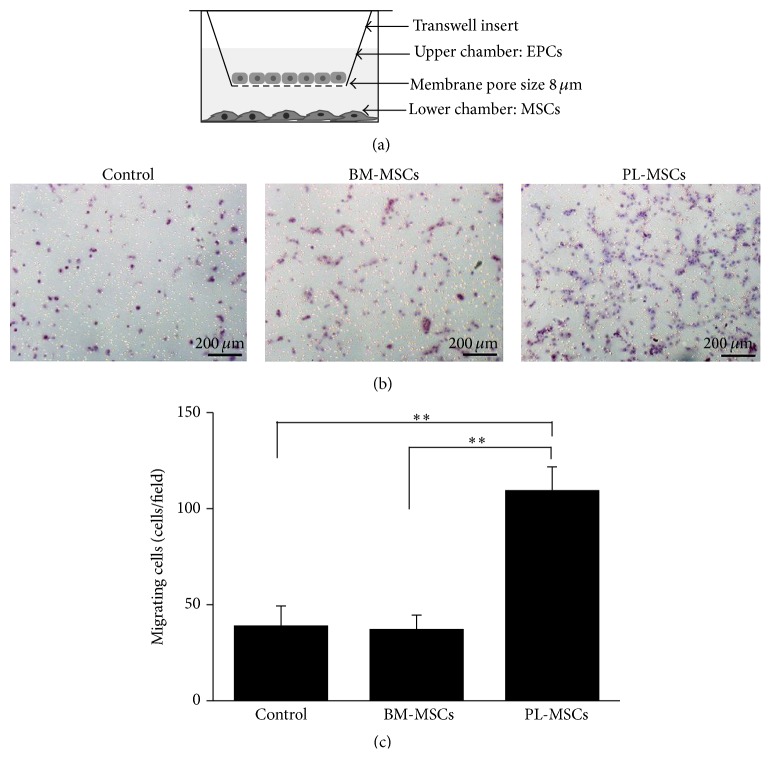
Effects of soluble factors released from BM-MSCs and PL-MSCs on EPC migration. (a) Diagram of the transwell culture system for an* in vitro* migration assay. (b) Hematoxylin stained EPCs which migrated to the other side of transwell membrane in response to soluble factors released from BM-MSCs and PL-MSCs. EPCs cultured in transwells containing serum-free medium (SFM) serve as negative controls. (c) Number of EPCs which migrated in response to soluble factors released from BM-MSCs and PL-MSCs. Data were presented as mean ± SEM of three independent experiments. Mann-Whitney *U* test was used to assess the significance of differences between observed data. ^*∗∗*^ *P* < 0.01.

To investigate the effect of MSC-derived soluble factors on EPC migration, EPCs were cocultured with BM-MSCs and PL-MSCs using the transwell culture system (Figure 3(a)). The numbers of EPCs that migrate toward BM-MSCs and PL-MSCs were determined after 6 hours of coculture. The soluble factors derived from PL-MSCs significantly enhanced EPC migration compared to controls (109.7 ± 12.0 cells/field versus 39.2 ± 10.1 cells/field, *P* = 0.002) while BM-MSCs failed to induce EPC migration (37.4 ± 7.2 cells/field versus 39.2 ± 10.1 cells/field of controls) (Figures 3(b) and 3(c)), suggesting that only soluble factors which were uniquely released from PL-MSCs could enhance EPC migration.

### 3.3. Effect of Fractionated PL-MSCs Conditioned Medium on EPC Migration

**Figure 4:**
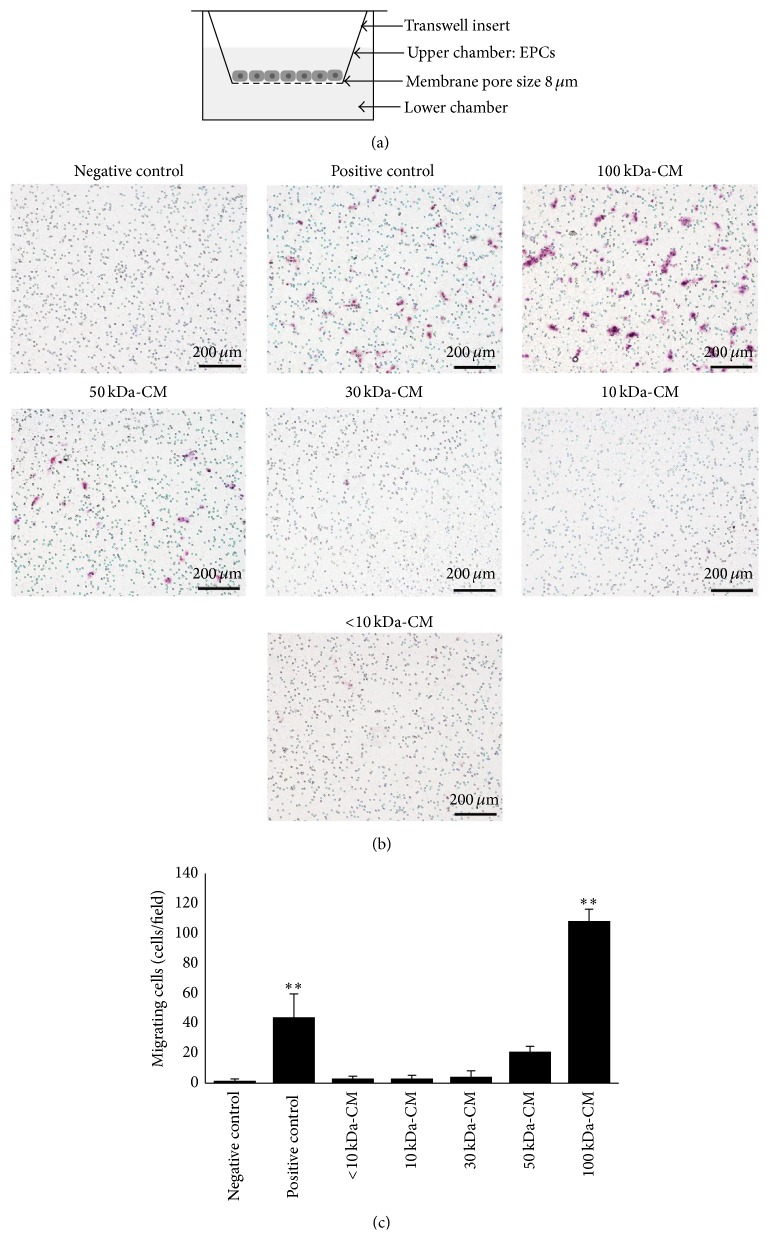
Effect of PL-MSCs conditioned medium fractions on EPC migration. (a) Diagram of the transwell culture system for an* in vitro* migration assay. (b) Hematoxylin stained EPCs which migrated to the other side of membrane in response to soluble factors presented in the 100 kDa, 50 kDa, 30 kDa, 10 kDa, and less than 10 kDa fractions of the PL-MSCs conditioned medium (scale bar = 200 *μ*m). (c) Numbers of EPCs which migrated in response to soluble factors presented in the 100 kDa, 50 kDa, 30 kDa, 10 kDa, and less than 10 kDa fractions of PL-MSCs conditioned medium. EPCs cultured in transwells whose lower chamber contained serum-free medium (SFM) served as negative controls while EPCs cultured in transwells whose lower chamber contained unfractionated PL-MSCs conditioned medium served as positive controls. Data were presented as mean ± SEM of three independent experiments. Nonparametric Kruskal-Wallis test was used to assess the significance of differences between observed data. ^*∗∗*^ *P* < 0.01 versus negative control.

To identify PL-MSC-derived factors which are able to induce EPC migration, PL-MSCs conditioned medium was fractionated into 5 distinct fractions according to the molecular weight of their protein compositions. The effect of each PL-MSCs conditioned medium fraction on EPC migration was then determined by* in vitro *migration assay (Figure 4(a)). Only the unfractionated and 100 kDa fraction of PL-MSCs conditioned medium could significantly enhance EPC migration compared to negative controls (44.0 ± 15.6 versus 1.7 ± 1.2 cells/field, *P* = 0.01 for the unfractionated PL-MSC conditioned medium, and 108.2 ± 7.9 versus 1.7 ± 1.2 cells/field, *P* = 0.01 for the 100 kDa fraction) while the other fractions including 50 kDa fraction (21.1 ± 3.5 versus 1.7 ± 1.2 cells/field), 30 kDa fraction (4.4 ± 3.9 versus 1.7 ± 1.2 cells/field), 10 kDa fraction (3.1 ± 2.1 versus 1.7 ± 1.2 cells/field), and less than 10 kDa fraction (3.2 ± 1.6 versus 1.7 ± 1.2 cells/field) did not enhance EPC migration (Figures 4(b) and 4(c)). The number of migrated EPCs induced by the 100 kDa fraction, which were approximately 2.5-fold higher than those induced by the unfractionated PL-MSCs conditioned medium (Figure 4(c)), clearly indicates that the PL-MSC-derived factors which are able to induce EPC migration were fractionated into the 100 kDa fraction.

### 3.4. Effects of the 100 kDa Subfractions of PL-MSCs Secretome on EPC Migration

**Figure 5:**
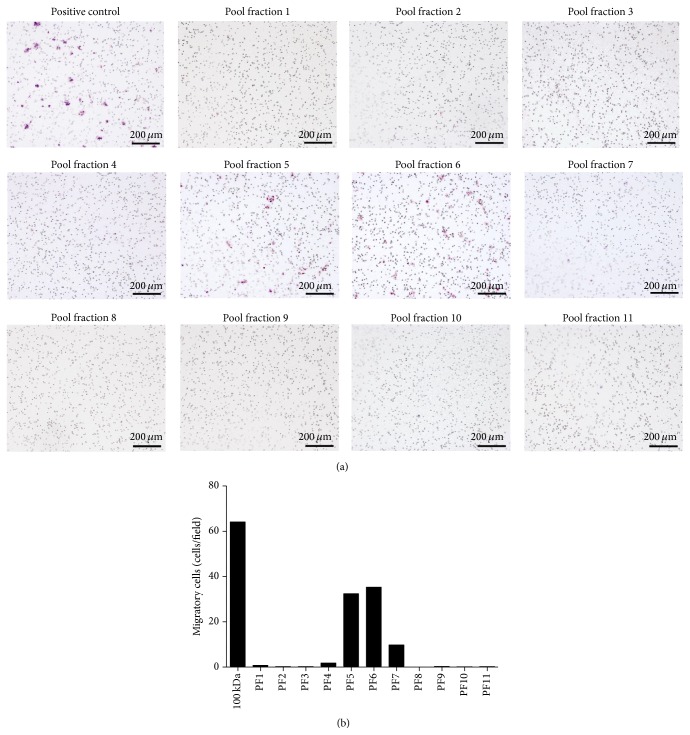
Effect of the 100 kDa subfractions on EPC migration. (a) Hematoxylin stained EPCs which migrated to the other side of membrane in response to soluble factors presented in 11 distinct subfractions (PF) of the 100 kDa fraction of PL-MSCs conditioned medium (scale bar = 200 *μ*m). (b) Number of EPCs which migrated in response to soluble factors presented in 11 distinct subfractions of the 100 kDa fraction of PL-MSCs conditioned medium.

To identify EPC migratory enhancing factors presented in the 100 kDa of PL-MSCs conditioned medium, proteins presented in the 100 kDa fraction of PL-MSCs conditioned medium were further fractionated into 11 subfractions according to their hydrophobicity (see Supplementary Figure 1 of the Supplementary Material available online at http://dx.doi.org/10.1155/2016/2514326). The effect of each 100 kDa subfraction on EPC migration was then determined by* in vitro* migration assay. Only subfractions 5 and 6 of the 100 kDa fraction could enhance EPC migration (32.4 cells/field and 35.32 cells/field, resp.) whereas the rest of the subfractions did not (Figures 5(a) and 5(b)). The numbers of migrated EPCs induced by subfractions 5 (32.4 cells/field) and 6 (35.32 cells/field) were 2-fold lower than those induced by the 100 kDa fraction (64.12 cells/field), indicating that subfraction 5 and subfraction 6 of the 100 kDa fraction contained EPC migratory enhancing factors.

### 3.5. Identification of Candidate EPC Migratory Enhancing Factors in PL-MSCs Secretome

**Figure 6:**
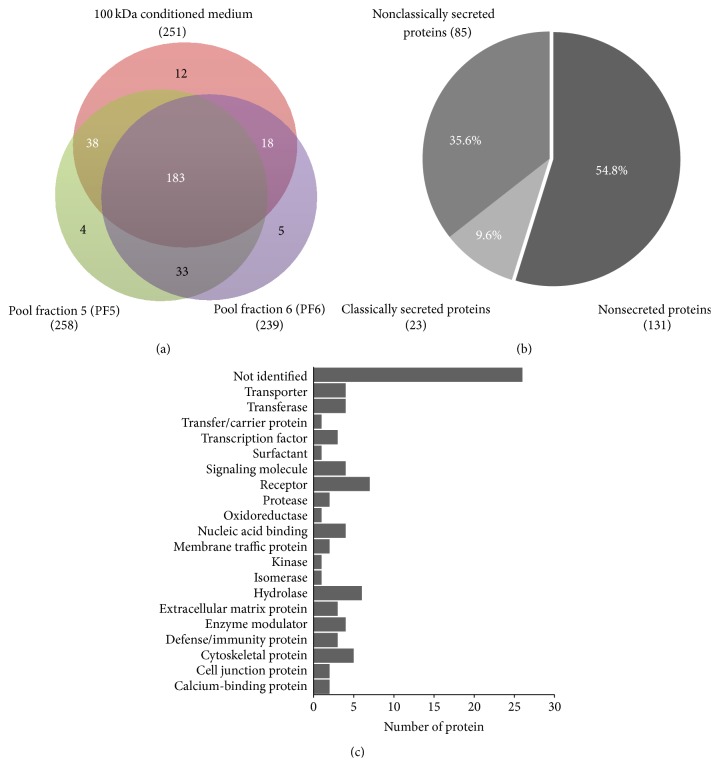
Characterization of PL-MSCs secretome. (a) Venn diagram illustrates the proteins presented in 100 kDa fraction of PL-MSCs conditioned medium as well as those presented in subfraction 5 and subfraction 6 of the 100 kDa fraction. Number of proteins presented in the 100 kDa fraction which were subsequently fractionated into subfraction 5 and/or subfraction 6 (labeled with white color) were regarded as candidate proteins. (b) Pie chart illustrates 239 candidate proteins which were further categorized into nonsecreted protein, classically secreted protein, and nonclassically secreted protein, by SignalP 4.1 and SecretomeP 2.0 software. (c) Graph demonstrates functions of 77 secretory proteins based on PANTHER classification and UniProt.

EPC migratory enhancing factors presented in PL-MSCs secretome were identified by mass spectrometry. Proteins presented in the 100 kDa fraction, subfraction 5, and subfraction 6 were identified by DeCyder differential analysis software based on their signal intensities. The 100 kDa fraction contained 251 proteins while subfraction 5 and subfraction 6 contained 258 and 239 proteins, respectively (Figure 6(a)). The proteins presented in 100 kDa fraction which were subsequently fractionated into subfraction 5 and/or subfraction 6 were considered to be possible candidates for EPC migratory inducing factor. There were 183 proteins in all 3 fractions. 38 proteins were presented in 100 kDa fraction and subfraction 5 and 18 proteins were presented in 100 kDa fraction and subfraction 6 (Figure 6(a)).

Those 239 (183 + 38 + 18) candidate proteins were further categorized into nonsecreted protein, classically secreted protein, and nonclassically secreted protein by SignalP 4.1 and SecretomeP 2.0 software. Of 239 proteins, 131 were nonsecreted proteins, 85 were nonclassically secreted proteins, and 23 were classically secreted proteins (Figure 6(b)). To identify EPC migratory enhancing factors, 162 nonsecreted and unidentified proteins were excluded while 77 nonclassically and classically secreted proteins (Supplementary Table 1) were further categorized into various groups based on their functions, such as extracellular matrix, receptors, proteinase, cytoskeletal proteins, cytokine, and enzymes (Figure 6(c)) by PANTHER classification and UniProt software.

Among those 77 secreted proteins, we further identified the possible candidates for EPC migratory enhancing factor using the following criteria: (A) the candidates must be classical secretory proteins, (B) the candidates must not belong to an apoptotic pathway, and (C) the candidates must be previously reported to be involved in cell migration and/or neovascularization process. According to those criteria, 12 proteins were considered to be possible candidates for EPC migratory enhancing factors. These proteins include astrotactin-1, ADAMTS1, plexin-B1, heparin cofactor 2, Sushi domain-containing protein 2, plasminogen (Angiostatin), PILR alpha-associated neural protein, lymphocyte antigen 75, type IV collagen, laminin, semaphorin receptor, and small inducible cytokine subfamily E, member 1 (endothelial monocyte-activating) (Table 1).

## 4. Discussion

MSCs have been regarded as promising sources for cell therapy. The therapeutic potentials of MSCs depend on their multilineage differentiation capacity and their ability to secrete broad range of cytokines and growth factors which modulate immune response, enhance cell survival, and induce neovascularization in the target tissues [20]. The secretome profiles of BM-MSCs and adipose tissue-derived MSCs (AD-MSCs) have been analyzed [18, 21] and were shown to contain several proangiogenic factors including VEGF, bFGF, ANGPT1, IL6, MCP-1, and SDF1 [22]. Furthermore, a previous study also demonstrated that factors released from mouse BM-MSCs enhanced proliferation, survival, migration, and vessel-forming capacity of mature endothelial cells [23]. However, the secretome profile of gestational tissue-derived MSCs and their effects on the functions of EPCs which play an important role during neovascularization is still poorly characterized.

We showed that PL-MSCs derived soluble factors significantly enhanced EPC migration and their migration enhancing effect was even greater than that of BM-MSCs. Despite the fact that there are several reports describing the positive effect of BM-MSCs on the function of mature endothelial cells (ECs), there have been no previous reports on the effects of BM-MSCs on the properties of endothelial progenitor cells (EPCs). Previous studies demonstrated the enhancing effect of BM-MSCs on EC migration; however our results showed that BM-MSCs did not affect EPC migration. It is possible that the EC and EPC migration requires different proangiogenic factors. Previous studies showed that VEGF released from BM-MSCs and chorionic blood vessel-derived MSCs (bv-MSCs) enhances EC migration [24–27]. In our study, a subfraction of secretome from PL-MSCs supposed to contain VEGF did not enhance EPC migration in the transwell culture system.

To further identify EPC migration enhancing factors presented in PL-MSCs secretome, PL-MSCs conditioned medium was separated into several distinct fractions according to the molecular weight and hydrophobicity of their protein components. EPC migration enhancing factors were enriched in subfractions 5 and 6 of the 100 kDa fraction of PL-MSCs secretome. Mass spectrometry analysis revealed that PL-MSCs secreted hundreds of different proteins through classical and nonclassical secretory pathway. The nonsecretory intracellular proteins leaked from dead cells during culture were also identified in the PL-MSCs conditioned medium and excluded from subsequent analysis [28].

Using this strategy, 77 PL-MSC-derived secretory proteins presented in subfraction 5 and/or 6 of the 100 kDa fraction of PL-MSCs secretome were identified. Among those secreted proteins, 12 proteins previously reported to be involved in cell migration and/or neovascularization process were identified as candidates for EPC migration enhancing factors. Those include (A) laminin and collagen IV which are extracellular matrix proteins involved in cell adhesion and migration of endothelial and tumor cells [29–31], (B) a disintegrin and metalloproteinase with thrombospondin motif (ADAMTS1), a member of metalloprotease family, which plays important roles in ECM degradation and cell migration; a previous study demonstrated that ADAMTS1 enhanced proliferation and migration of endothelial cells under hypoxic condition [32], (C) a protein semaphorin and its receptor plexin-B1 which possesses proangiogenic activity [33], (D) serpin peptidase inhibitor, clade D, member 1 (SERPIND1) which promotes angiogenesis by enhancing proliferation, migration, and vessel-forming capacity of endothelial cells [34], and (E) angiostatin and endothelial monocyte-activating polypeptide II which exerts its EPC migration inducing effect through its receptor CXCR3 [35, 36].

The positive effect of PL-MSCs on EPC migration described in our study is in agreement with a previous report demonstrating that several gestational tissue-derived MSCs, including chorionic blood vessel-derived MSCs (bv-MSCs), amniotic membrane-derived MSCs (hAMCs), and umbilical cord-derived MSCs (UC-MSCs) released proangiogenic factors which enhance migration and vessel-forming capacity of mature endothelial cell [24, 27, 37]. We have identified several novel proteins in addition to the well-known proangiogenic factors described in previous studies using ELISA array to identify MSC secretome [24, 27, 37]. The mass spectrometry analysis used in our study could discover the complete protein composition of PL-MSCs secretome in an unbias manner and with higher sensitivity while an ELISA array can only determine the amount of limited number of well-known proangiogenic factors presented in PL-MSCs. Our study is also the first report that describes the effects of PL-MSCs secretome on the migration capacity of EPCs.

## 5. Conclusion

We herein report for the first time that PL-MSCs secreted unique combination of factors which enhance EPC migration and their effect is greater than that of BM-MSCs. We also identified several novel candidates for EPC migration enhancing factor in PL-MSCs secretome which have been reported to enhance proliferation, migration, and vessel-forming capacity of mature endothelial cells. The factors discovered in this study could be used to implement the therapeutic effect of MSCs by enhancing neovascularization in the injured/ischemic tissues. Moreover, the strategy developed in our study could be used to systematically identify other therapeutically useful molecules in the secretomes of other MSC sources. However, the therapeutic effects of EPC migration enhancing factors identified in this study should be further confirmed by* in vitro* and* in vivo* studies before use in the clinical applications.

## Supplementary Material

Figure 1: Quantity of total proteins presented in each subfraction of the 100 kDa fraction of PL-MSC secretome.
The amounts of total proteins and peptides presented in each subfraction of the 100 kDa fraction of PL-MSC secretome as determined by measuring an absorbance at 280 nm (to detect proteins containing aromatic ring) and 220 nm (to detect peptides). Each subfraction of 100 kDa fraction was pooled as follow:
Subfraction 1 (PF1) is the proteins collected at 1 minute to 14 minutes.Subfraction 2 (PF2) is the proteins collected at 15 minutes to 20 minutes.Subfraction 3 (PF3) is the proteins collected at 21 minutes to 29 minutes.Subfraction 4 (PF4) is the proteins collected at 30 minutes to 34 minutes.Subfraction 5 (PF5) is the proteins collected at 35 minutes to 43 minutes.Subfraction 6 (PF6) is the proteins collected at 44 minutes to 51 minutes.Subfraction 7 (PF7) is the proteins collected at 52 minutes to 60 minutes.Subfraction 8 (PF8) is the proteins collected at 61 minutes to 70 minutes.Subfraction 9 (PF9) is the proteins collected at 71 minutes to 80 minutes.Subfraction 10 (PF10) is the proteins collected at 81 minutes to 90 minutes.Subfraction 11 (PF11) is the proteins collected at 91 minutes to 100 minutes.
Supplementary table 1: List of 77 secreted proteins identified in PL-MSC secretome.

## Figures and Tables

**Table 1:** List of EPC migratory enhancing factor candidates in PL-MSCs secretome.

Protein name	Accession number	Secretory pathway prediction	Biological function	UniProtKB accession number
Astrotactin-1	gi|46488923	Classical	Neuron migration and neuronal adhesion	O14525
ADAMTS1	gi|119631213	Classical	Metalloprotease involve in extracellular matrix remodeling	Q9UHI8
Plexin-B1	gi|6010211	Classical	Axon guidance and cell migration	O43157
Heparin cofactor 2 (SERPIND1)	gi|23273330	Classical	Chemotactic activity for monocyte and neutrophil	P05546
Sushi domain-containing protein 2	gi|10092665	Classical	Immune response	Q9UGT4
Plasminogen (angiostatin)	gi|38051823	Classical	Blood coagulation Angiogenesis inhibitor	P00747
PILR alpha-associated neural protein	gi|24308547	Classical	Immune regulation	Q8IYJ0
Lymphocyte antigen 75	gi|32307817	Classical	Inflammation and immune process	O60449
Type IV collagen	gi|15991848	Classical	Extracellular matrix/cell adhesion	Q14031
Laminin	gi|119613854	Classical	Cell adhesion and cell migration	Q13751
Semaphorin receptor	gi|6010211	Classical	Axon guidance and cell migration	O43157
Small inducible cytokine subfamily E, member 1 (endothelial monocyte-activating)	gi|119626608	Nonclassical	Angiogenesis	Q12904
