# Supplementary material for: Endothelial Progenitor Cell Migration-Enhancing Factors in the Secretome of Placental-Derived Mesenchymal Stem Cells
Source: Stem Cells Int. 2016 Jan 6;2016:2514326. doi: 10.1155/2016/2514326 (PMC4736766; doi:10.1155/2016/2514326)
Supplement: Supplementary file 1 — Figure 1: Quantity of total proteins presented in each subfraction of the 100 kDa fraction of PL-MSC secretome. The amounts of total proteins and peptides presented in each subfraction of the 100 kDa fraction of PL-MSC secretome as determined by measuring an absorbance at 280 nm (to detect proteins containing aromatic ring) and 220 nm (to detect peptides). Each subfraction of 100 kDa fraction was pooled as follow: Subfraction 1 (PF1) is the proteins collected at 1 minute to 14 minutes.Subfraction 2 (PF2) is the proteins collected at 15 minutes to 20 minutes.Subfraction 3 (PF3) is the proteins collected at 21 minutes to 29 minutes.Subfraction 4 (PF4) is the proteins collected at 30 minutes to 34 minutes.Subfraction 5 (PF5) is the proteins collected at 35 minutes to 43 minutes.Subfraction 6 (PF6) is the proteins collected at 44 minutes to 51 minutes.Subfraction 7 (PF7) is the proteins collected at 52 minutes to 60 minutes.Subfraction 8 (PF8) is the proteins collected at 61 minutes to 70 minutes.Subfraction 9 (PF9) is the proteins collected at 71 minutes to 80 minutes.Subfraction 10 (PF10) is the proteins collected at 81 minutes to 90 minutes.Subfraction 11 (PF11) is the proteins collected at 91 minutes to 100 minutes. Supplementary table 1: List of 77 secreted proteins identified in PL-MSC secretome. [file 2514326.f1.pdf]

Supplementary information

Supplementary figure 1:

The amounts of total proteins and peptides presented in each subfraction of the 100kDa fraction of PL-MSC conditioned medium as determined by measuring an absorbance at 280 nm (to detect proteins containing aromatic ring) and 220 nm (to detect peptides)

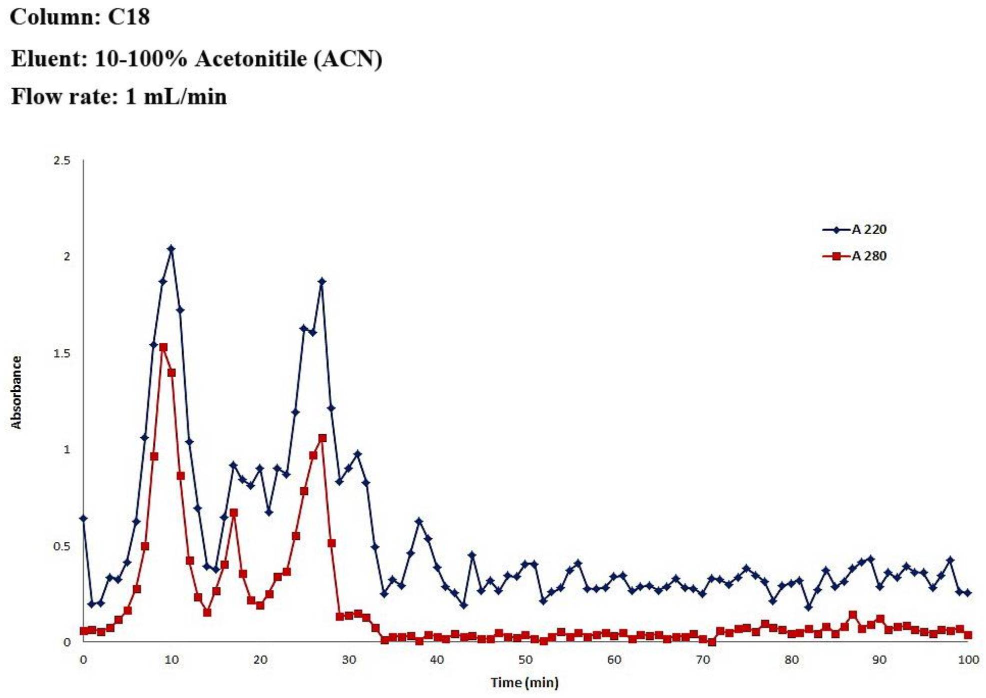

Supplementary table 1: List of 77 secreted proteins identified in PL-MSC secretome

Protein name	Accession number	SignalP	SecretomeP	PANTHER classification
Astrotactin-1	gi|46488923	Yes		Cell junction protein
Heparan-sulfate 6-O-sulfotransferase 2 isoform L	gi|116295256		Yes	Transferase
Motile sperm domain-containing protein 1	gi|9506543		Yes	-
Phospholipid-transporting ATPase IB	gi|117168245		Yes	Hydrolase<br>Transporter
Polyamine-modulated factor 1 isoform 3	gi|313851053		Yes	-
RCSD1 protein	gi|48735391		Yes	Cytoskeletal protein
SLIT-ROBO Rho GTPase activating protein 2	gi|55662261		Yes	-
A type IV collagen	gi|15991848	Yes		Extracellular matrix
ADAM metallopeptidase with thrombospondin type 1 motif	gi|119631213	Yes		Protease
Antigen MLAA-21	gi|37574654		Yes	Cell junction protein<br>G-protein modulator
Apical protein 2, isoform CRA_a	gi|119582714		Yes	-
Arylsulfatase D precursor	gi|71852584	Yes		Hydrolase
B-cell CLL/lymphoma 7 protein family member B isoform 2	gi|308522773		Yes	-
C1q and tumor necrosis factor related protein 3 variant	gi|62088102		Yes	-
C3 and PZP-like alpha-2-macroglobulin domain containing 8	gi|23379807	Yes		Signaling molecule
CD95 ligand (Tumor necrosis factor)	gi|61658441		Yes	-
Chain A, Crystal Structure Of The Mammalian Copii-coat Protein Sec23/24 Bound To The Transport Signal Sequence Of Membrin	gi|209870512		Yes	G-protein modulator
Chain A, Crystallographic And Kinetic Studies Of Human Mitochondrial Acetoacetyl-coa Thiolase (t2): The Importance Of Potassium And Chloride For Its Structure And Function	gi|145579614		Yes	Acetyltransferase
Chain A, Hr1b Domain From Prk1	gi|159163170		Yes	Kinase<br>Carrier protein
Chain A, Solution Structure Of The Death Domain Of Ankyrin-1	gi|171848938		Yes	Cytoskeletal protein
Chain A, The Three-Dimensional Structure Of The C-Terminal Dna Binding Domain Of Human Ku70	gi|15988053		Yes	Nucleic acid binding
Chain A, X-Ray Structure Of Human Phosphomannomutase 2 (Pmm2)	gi|75766280		Yes	Isomerase
Chain B, Crystal Structure Of The Human Taf4-Taf12 (Tafii135-Tafii20) Complex	gi|24158663		Yes	-
Chaperonin containing TCP1, subunit 4 (delta), isoform CRA_a	gi|119620390		Yes	Nucleotide binding
Chondroitin sulfate synthase 1 precursor	gi|31542309	Yes		Glycosyltransferase

Supplementary table 1: List of 77 secreted proteins identified in PL-MSC secretome (cont.)

Protein name	Accession number	SignalP	SecretomeP	PANTHER classification
Coiled-coil domain containing 78, isoform CRA_f	gi|119606147		Yes	-
Cystine/glutamate transporter	gi|7657683		Yes	Amino acid transporter
DEC-205/DCL-1 fusion protein variant V34-2	gi|32307817	Yes		Receptor
Delta-like protein 1 precursor	gi|110735443	Yes		Cell signaling
Down syndrome cell adhesion molecule isoform CHD2-42 precursor variant	gi|62087852	Yes		-
EP3-V	gi|2114191		Yes	G-protein coupled receptor
ERI1 exoribonuclease 3 isoform 1	gi|74136559		Yes	Hydrolase
General transcription factor IIF, polypeptide 1, 74kDa, isoform CRA_b	gi|119589505		Yes	Transcription factor binding
HEATR1 protein	gi|15080480		Yes	-
Histidine triad nucleotide binding protein 2, isoform CRA_c	gi|119578737		Yes	-
Hkir2.2x	gi|48474202		Yes	Ion transport
Ig kappa chain V-III region (Sca) - human (fragment)	gi|106611		Yes	-
Immunoglobulin gamma 2 heavy chain variable region	gi|304562513		Yes	-
Immunoglobulin kappa light chain variable region	gi|116795057		Yes	-
Immunoglobulin lambda chain variable region	gi|587390		Yes	-
Immunoglobulin light chain variable region	gi|13549148		Yes	-
Immunoglobulin variable region	gi|323431845		Yes	-
KIAA0391	gi|27882031		Yes	-
KIAA0976 protein	gi|40789006		Yes	Receptor/ ECM linker protein
KIF6 protein	gi|109658866		Yes	Cytoskeletal protein
Laminin, beta 3, isoform CRA_a	gi|119613854	Yes		ECM linker protein
LDLR-FUT fusion protein	gi|6739500	Yes		-
Leprecan-like 2, isoform CRA_c	gi|119609139		Yes	Extracellular matrix organization
MHC class II antigen	gi|84796223	Yes		Immunity
MSTP018 (Integrin alpha-11)	gi|17432223		Yes	Cell adhesion/Receptor
Mutant CD8 alpha antigen	gi|14861040	Yes		Defense/immunity protein
Nebulette	gi|3660517		Yes	-
NK1 transcription factor-related protein 2	gi|226437602		Yes	Nucleic acid binding<br>Transcription factor

Supplementary table 1: List of 77 secreted proteins identified in PL-MSC secretome (cont.)

Protein name	Accession number	SignalP	SecretomeP	PANTHER classification
Phosphodiesterase isozyme 7	gi|30421104		Yes	Signal transduction
PILR alpha-associated neural protein isoform a precursor	gi|24308547	Yes		Immunity molecule
Plasminogen	gi|38051823	Yes		Peptide hormone/receptor/serine proteinase
Pyruvate dehydrogenase phosphatase regulatory subunit	gi|152013038		Yes	Oxidoreductase
RecName: Full=Zinc finger and BTB domain-containing protein 44	gi|74760158		Yes	Transcription cofactors
SEMA6C protein	gi|92058719	Yes		Signaling molecule
Semaphorin receptor	gi|6010211	Yes		Receptor
Serpin peptidase inhibitor, clade D (heparin cofactor)	gi|23273330	Yes		Hydrolase activity
Serum amyloid A protein beta des-Arg(pI5.6), SAA1 beta des-Arg pI5.6	gi|247142		Yes	-
SETMAR protein, partial	gi|33869529		Yes	DNA binding
Seven transmembrane helix receptor	gi|21928448		Yes	-
Small inducible cytokine subfamily E, member 1 (endothelial monocyte-activating), isoform CRA_a	gi|119626608		Yes	Cytokine activity
Solute carrier family 6, member 15, isoform CRA_a	gi|119617794		Yes	Ion transport
Sushi domain-containing protein 2 precursor	gi|10092665	Yes		Immnunity/cytokine
Synaptotagmin-10	gi|39752671		Yes	Membrane trafficking regulatory protein
Syntaxin 16	gi|2961087		Yes	Membrane trafficking regulatory protein
TAF15	gi|1373378		Yes	-
TBC1 domain family, member 7 variant	gi|62898451		Yes	-
Tumor necrosis factor receptor superfamily member 8 isoform 1 precursor	gi|597709795	Yes		Receptor
Ubiquitously transcribed tetratricopeptide repeat protein Y-linked transcript variant 283	gi|151946833		Yes	Transcription factor
Ubiquitously transcribed tetratricopeptide repeat protein Y-linked transcript variant 4	gi|148733168		Yes	Transcription factor
Voltage-gated calcium channel alpha(2)delta-4 subunit	gi|22770594		Yes	Calcium-binding protein
WD repeat domain 35	gi|22477171		Yes	-
Zinc finger protein 449	gi|194239638		Yes	Nucleic acid binding
